# Cell-Free Fermentation Broth of *Bacillus velezensis* Strain S3-1 Improves Pak Choi Nutritional Quality and Changes the Bacterial Community Structure of the Rhizosphere Soil

**DOI:** 10.3389/fmicb.2020.02043

**Published:** 2020-09-10

**Authors:** Kaiheng Lu, Qing Jin, Yibo Lin, Wenwei Lu, Songshuo Li, Chenhao Zhou, Jieren Jin, Qiuyan Jiang, Lichen Ling, Ming Xiao

**Affiliations:** Shanghai Engineering and Technical Research Center of Plant Germplasm Resources, College of Life Sciences, Shanghai Normal University, Shanghai, China

**Keywords:** *Bacillus velezensis* strain S3-1, Pak choi, soil properties, bacterial community, soil enzyme activity

## Abstract

*Bacillus velezensis* is a plant growth-promoting rhizobacteria (PGPR) that has long been proven to improve the growth of plants, and it has been widely used in agriculture. However, in many reports, we observed that during the application of bacterial fluids, it appeared that the effect of the cell-free fermentation broth (CFB) was ignored. The purpose of this study is to compare the effect of the no inoculation treatment (CK), the *B. velezensis* strain S3-1 treatment (S), the CFB treatment in the Pak choi, soil bacterial community structure, soil enzyme activity, and field soil properties. The results have shown that, compared to the inoculation *B. velezensis* strain S3-1 treatment and the no-inoculation treatment; the inoculation of the CFB treatment can significantly enhance the soluble protein, soluble solids, ascorbic acid of Pak choi and increase the total phosphorus content and electrical conductivity (EC) in the soil. Based on high-throughput sequencing data, our analysis of soil microbial communities used R, NETWORK, and PICRUSt showed that the CFB treatment can enhance the relative abundance of *Acidobacteria* in the soil, decrease the abundance of native *Bacillus* in the soil, change the microbial community structure of the top 50 operational taxonomic units (OTUs), and improve soil microbial carbon metabolism and nitrogen metabolism. Overall, we observed that CFB treatment can also improve plant nutrition and change soil microbial communities. This study provides new insights for the application of microbial fertilizers in agricultural production.

## Introduction

Pak choi (*Brassica campestris* L. ssp. *chinensis* Makino) is one of the most widely used and commercially essential crops in China. It has high nutritional value as it provides an intake of cellulose, vitamins, and minerals (Han et al., 2019). Therefore, there is great interest in improving Pak choi nutritional quality by using rhizosphere-promoting bacteria.

The use of the plant growth-promoting rhizobacteria (PGPR) as a substitute minimizes the adverse effects of artificial fertilizers and pesticides on the environment. Compared to the use of chemical fertilizers, bio-fertilizers made from rhizosphere bacteria can also provide better yield and nutritional value for organic fruits and Pak choi (Al-Farsi and Lee, 2008).

PGPR can be used as bio-fertilizers. They are applied to the soil to support the availability and absorption of nutrients, especially under adverse conditions (Hamad et al., 2015). In the soil, PGPR inoculants have been reported to participate in nutrient cycling and increase crop productivity (Singh et al., 2011). *Bacillus velezensis* is widely reported to be in the soil and plants as an essential plant rhizosphere-promoting bacteria (Mateus et al., 2019; Rabbee et al., 2019). *B. velezensis* can produce volatile organic compounds (VOCs), iturin, fengycin, surfactin, and antibiotics, among others, to inhibit fungal growth (Jin et al., 2017). Bacteria can also inhibit the growth of fungi by altering the gene expression of the fungus (Kang et al., 2019). At the same time, bacteria can produce indole acetic acid (IAA), siderophore, and phosphorus solubilization to promote plant growth (Kim et al., 2017). For example, *B. velezensis* GF267 is capable of producing siderophore that increases the chlorophyll content of tomatoes (Paula et al., 2019), and *B. velezensis* also promotes soybean and *Brassica* growth (Kanjanamaneesathian et al., 2013; Hassan et al., 2019). However, the survival of the bio-fertilizers in the soil tends to be poor due to the complexity of the soil environment. Furthermore, the role played by cell-free fermentation broth (CFB) is unclear (Wang et al., 2019).

Soil rhizosphere microbial communities play an important role in plant growth. For instance, different fertilization treatments change soil microbial communities and affect apples growth (Thompson et al., 2019). Moreover, soil microbial communities improve the resistance to the invasion of fungi (Li et al., 2019; Wei et al., 2019). Nevertheless, few studies have focused on the link between CFB and soil microbial communities.

Here, we studied the properties of *B. velezensis* S3-1, a strain isolated from the cucumber rhizosphere soil. The whole-genome sequencing results of this strain indicate that it has the ability to produce substances, such as IAA and 1-aminocyclopropane-1-carboxylate (ACC) deaminase, and has the potential to promote plant growth (Jin, 2019). In the present study, we aimed to understand (i) how CFB affects Pak choi nutritional quality; (ii) the relationship between the effects of CFB on soil properties and Pak choi nutritional quality; and (iii) the relationship between the effects of CFB on the rhizosphere soil microbial community.

## Materials and Methods

### Preparation of Inoculum and Identification of Fermentation Products

*B. velezensis* S3-1 (CCTCC AB 2014337) was grown in Luria-Bertani (LB) medium (10 g/L Tryptone, 5 g/L yeast extract, and 5 g/L NaCl) at 28°C (with shaking at 200 rpm) until the number of 5 × 10^8^ CFU ml^−1^. Then the CFB was clarified by centrifugation at 10,000 *g* for 20 min at 4°C. The bacterial inoculum was prepared by centrifugation at 3,000 *g* for 20 min at 4°C and resuspension of the pellet in equal volumes of water.

Take 200 μl of CFB and add 500 μl of methanol: acetonitrile = 2:1 (containing 5% of internal standard L-2-chloro-phenylalanine), vortex for 30 s; after vortex mixing, ice-water bath ultrasonic extraction 30 min; let the sample stand at −20°C, 30 min; centrifuge at 4°C 13,000 rcf for 15 min; take the supernatant, put it into a glass derivatization bottle, and blow dry with nitrogen; add 80 μl of methoxyamine hydrochloride to the glass derivation vial Pyridine solution (15 mg/ml), vortex and shake for 2 min, then perform oxime reaction in a shaking incubator at 37°C for 90 min; remove the sample and add 80 μl of BSTFA (containing 1% TMCS) derivative reagent, vortex and shake for 2 min, and react at 70°C for 60 min; and after removing the sample, leave it at room temperature for 30 min for GC-MS metabolomics analysis.

After derivatization, the sample was injected into the GC-MS system in splitless mode for analysis, and the injection volume was 1 μl. The sample was separated by HP-5MS UI capillary column (30 m × 0.25 mm × 0.25 μm, Agilent J&W Scientific, Agilent 19091S-433), and then subjected to mass spectrometry. The inlet temperature is 260°C, high-purity helium is used as the carrier gas, the carrier gas flow rate is 1 ml/min, the septum purge flow rate is 3 ml/min, and the solvent is delayed by 5 min. Heating program: initial temperature 60°C, equilibration for 0.5 min, then increase to 310°C at a rate of 8°C/min, and maintain for 6 min. The electron bombards the ion source (EI), the transmission line temperature is 310°C, the ion source temperature is 230°C, the quadrupole temperature is 150°C, and the electron energy is 70 eV. Scanning mode is full scan mode (SCAN), mass scanning range: m/z 50–500, scanning frequency is 3.2 scan/s.

### Characterization of Bacteria

One milliliter the bacterial suspension (5 × 10^8^ CFU ml^−1^) was inoculated into 100 ml of LB broth containing L-tryptophan (100 μg ml^−1^), and then the mix was incubated at 28°C for 7 days, while estimating the concentration of IAA in the culture supernatant every day as described in the literature (Bano and Musarrat, 2003). The enzymatic activity of ACC deaminase was measured as described previously (Penrose and Glick, 2003). The phosphate solubilizing activity of the bacterial isolate was determined using Pikovskaya agar containing precipitated tricalcium phosphate (Pikovskaya, 1948). If a transparent area is observed, bacteria are considered to have the ability to dissolve phosphorus. Chitinase was detected by the colloidal chitin medium using the method of Frändberg and Schnürer (Emma and Johan, 1998). Detection of proteases was determined as described in the literature (Dutta et al., 2015). The cellulose degradation ability of the bacterial isolate was analyzed by streaking on a cellulose Congo red agar medium (Gupta et al., 2012). The lipase activity of the bacterial isolate was determined using a Tween lipase indicator assay. The appearance of a transparent circle is considered to be an indication of lipase activity (Howe and Ward, 1976). Chrome azurol S (CAS) agar media were used for the evaluation of siderophores production (Alexander and Zuberer, 1991).

### Plant Growth Stimulation With *B. velezensis* S3-1

To test the plant growth-promoting ability and optimal concentration of *B. velezensis* strain S3-1, the seeds of Pak choi were surface-sterilized with a 1% NaClO solution for 5 min, sterilized with 95% ethanol for 3 min, washed five times with sterile distilled water (Egamberdieva et al., 2017), placed on a sterile filter paper in a Petri dish, inoculated with 10 ml of different concentrations of *B. velezensis* strain S3-1 fermentation broth (based on dilution of one culture), 100(5 × 10^8^ CFU ml^−1^), 10(5 × 10^7^ CFU ml^−1^), 4(2 × 10^7^ CFU ml^−1^), 2(1 × 10^7^ CFU ml^−1^), and 1%(5 × 10^6^ CFU ml^−1^), with 10 seeds in each treatment, in triplicate. The seeds were grown in a plant growth chamber for 8 h at 28°C and then 16 h at 22°C in the dark. The root length and stem length of the plants were measured after 7 days.

### Field Experimental Design

The field trials were carried out in the area of Shanghai city. The mean temperature of the growing season in 2018 was 13–19°C (October to November) and 19–25°C (October to November). The pH of the soil was 7.37 and EC = 1,143. The experimental plots (1.5 m × 3.5 m) were arranged in a randomized block design with three replicates per treatment. This experiment lasted 49 days. Seeds were sown by hand (about 10 g per field). The three treatments were as follows: (i) no inoculation control (CK), (ii) plant inoculated with the bacterial inoculum mixed with water at a ratio of 1:100 (S), and (iii) plant inoculated with CFB mixed with water at a ratio of 1:100. The above treatments were used to water the fields twice a week, each field receiving about 10 L.

### Plant Physiology Analysis

The hydrogen peroxide (H_2_O_2_) content in Pak choi was evaluated as described by Mukherjee and Choudhuri (1983). Pre-cooled acetone at 4°C was used to extract the leaf samples (g: *V* = 1:1), and 1 ml of the supernatant was mixed with 0.1 ml of 5% TiSO_4_ and 0.2 ml of NH_4_OH (20%). The supernatant was removed by centrifugation. The precipitate was mixed with 5 ml of 2 mol H_2_SO_4_, centrifuged at 6,000 *g* for 10 min, and the supernatant was then read at 415 nm.

The titratable acid was titrated with 0.1 mol/L NaOH (Khaliq et al., 2019). Ascorbic acid was titrated with 0.1 g/L of 2,6-Dichlorophenol (Khaliq et al., 2019). For the soluble solids determination, an Abbe refractometer was used. Soluble proteins were determined using Coomassie Brilliant Blue G-250. Briefly, 1 ml of the supernatant was mixed with 5 ml of Coomassie Brilliant Blue G-250, incubated for 2 min, and read at 595 nm (7,504 UV/VIS Spectrometer, China; Bradford, 1976). The soluble sugar was determined using anthrone. A 0.5 ml sample extract was pipetted and mixed with 1 ml of distilled water, then 0.5 ml of anthrone-ethyl acetate and 5 ml of concentrated sulfuric acid were added, heated in 100°C water for 1 min, and naturally cooled and read at 630 nm (7,504 UV/VIS Spectrometer, China; Roman, 2002).

Plant roots were measured using a root scanner. Plant biomass was evaluated dry for constant weight at 105°C.

### Soil Sample Properties Analysis

The bulk soil was collected by shaking the roots of the plants, while the rhizosphere soil was collected by brushing (Chen et al., 2016). Six Pak choi were taken from each plot to collect rhizosphere soil for mixing. The rhizosphere soil was placed in coolers with ice packs and transported to the lab, where samples were kept at −80°C until further analysis.

One gram of rhizosphere soil was dried at 40–50°C, ground into a powder, passed through a 100-mesh sieve, and then analyzed for nitrogen (N) and carbon (C) with an elemental analyzer (ECS 8020, Italy; Howard et al., 2014). The dried soil was digested with aqua regia, and the elemental contents were analyzed using inductively coupled plasma optical emission spectrometry (ICP-OES; AcmeLabs, Canada; Zhang et al., 2017).

The bulk soil amylase and sucrase activities were detected using 3,5-dinitrosalicylic acid. For example, 5 g of soil were mixed with 15 ml of an 8% sucrose solution, 5 ml of phosphate buffer (pH = 5.5), and five drops of toluene, and then placed at 37°C for 24 h, heated in a boiling water bath for 5 min, and after cooling, it was read at 508 nm (Ge et al., 2017). The soil peroxidase and polyphenol oxidase activities were detected using 3,5-dinitrosalicylic acid. Briefly, 1 g of soil was mixed with 10 ml of 1% pyrogallol and 2 ml of 0.5% H_2_O_2_, and incubated in a 30°C incubator for 2 h. Then, 4 ml of citrate phosphate buffer (pH = 4.5) and 35 ml of ether were added. After an extraction period of 30 min, the samples were read at 430 nm (7504 UV/VIS Spectrometer, China). A standard colorimetric assay determined the soil urease activity (Kizilkaya, 2009).

### DNA Extraction and MiSeq Sequencing of 16S Amplicons

The rhizosphere soil DNA was extracted with the E.Z.N.A.® soil DNA Kit (Omega Bio-tek, Norcross, GA, United States). The DNA extract was checked on a 1% agarose gel, and the DNA concentration and purity were determined with NanoDrop 2000 UV-vis spectrophotometer (Thermo Scientific, Wilmington, United States). The hypervariable region V3-V4 of the bacterial 16S ribosomal RNA (rRNA) gene was amplified with primer pairs 338F (5′-ACTCCTACGGGAGGCAGCAG-3′) and 806R (5′-GGACTACHVGGGTWTCTAAT-3′) using an ABI GeneAmp® 9,700 PCR thermocycler (ABI, CA, United States). The PCR amplification of the 16S rRNA gene was performed as follows: initial denaturation at 95°C for 3 min, followed by 27 cycles of denaturing at 95°C for 30 s, annealing at 55°C for 30 s and extension at 72°C for 45 s, and then a single extension at 72°C for 10 min. The PCR mixtures contained 5 × TransStart FastPfu buffer 4 μl, 2.5 mM dNTPs 2 μl, forward primer (5 μM) 0.8 μl, reverse primer (5 μM) 0.8 μl, TransStart FastPfu DNA Polymerase 0.4 μl, template DNA 10 ng, and finally ddH_2_O up to 20 μl. PCR reactions were performed in triplicate. The PCR product was extracted from a 2% agarose gel and purified using the AxyPrep DNA Gel Extraction Kit (Axygen Biosciences, Union City, CA, Unites States) according to manufacturer’s instructions and quantified using Quantus™ Fluorometer (Promega, United States). In total, nine samples were prepared for sequencing with the MiSeq PE300 platform. The raw reads were deposited in the NCBI Sequence Read Archive (SRA:PRJNA534410) database.

### Data Analysis

The raw 16S rRNA gene sequencing reads were demultiplexed, quality-filtered by Trimmomatic, and merged by FLASH with the following criteria: (i) the 300 bp reads were truncated at any site receiving an average quality score of <20 over a 50 bp sliding window, and the truncated reads shorter than 50 bp were discarded, reads containing ambiguous characters were also discarded; (ii) only overlapping sequences longer than 10 bp were assembled according to their overlapped sequence. The maximum mismatch ratio of the overlap region was set to be 0.2. Reads that could not be assembled were discarded; and (iii) samples were distinguished according to the barcode (exact barcode matching) and primers (up to two nucleotide mismatches allowed), and the sequence direction was adjusted.

Operational taxonomic units (OTUs) with 97% similarity cutoff were clustered using UPARSE (version 7.1^1^), and chimeric sequences were identified and removed. The taxonomy of each OTU representative sequence was analyzed by RDP Classifier^2^ against the 16S rRNA database (e.g., Silva SSU128) using a confidence threshold of 0.7.

All of the statistical analyses were performed using R packages (V.3.5.1), in addition to using Networkx to analyze the mesh and constructing a phylogenetic tree using interactive tree of life (ITOL; Beckers et al., 2017). The variance analysis was performed using an LSD in SPSS 24.0 (SPSS Institute, USA). Values of *p* < 0.05 were considered to be significant.

The 16S function prediction was employed to standardize the OTU abundance table by PICRUSt, which was used to remove the effect of the number of copies of the 16S marker gene in the species genome. Manually screen out genes related to the C and N cycle, and the abundance of each gene could be calculated according to the OTU abundance.

## Results

### Identification of Fermentation Products and Characterization of the Bacteria

*B. velezensis* S3-1 could produce IAA (19.156 g/ml), ACC deaminase, phosphate solubilizing activity, and siderophores, but it was unable to produce lipase, chitinase, and cellulase (Table 1).

**Table 1:** Characterization of *B. velezensis* strain S3-1.

Strain	IAA (μg/ml)	Lipase	Protease	Chitinase	ACC deaminase	Phosphorus	Siderophore	Cellulase
S3-1	19.156	−	+	−	+	+	+	−

+, positive; −, negative.

**Figure 1:**
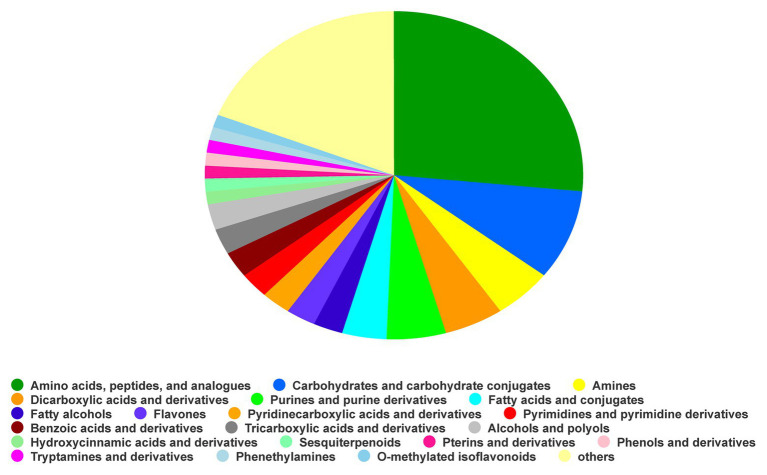
Compound classification based on HDBM.

Based on the NIST database for comparison, the identification results with a matching degree higher than 85 were considered to be reliable. A total of 192 substances were identified, which were divided into 20 types of substances others (18.99%), O-methylatedisoflavonoids (1.27%), phenethylamines (1.27%), tryptamines and derivatives (1.27%), phenols and derivatives (1.27%), pterins and derivatives (1.27%), sesquiterpenoids (1.27%), hydroxycinnamic acids and derivatives (1.27%), alcohols and polyols (2.53%), tricarboxylic acids and derivatives (2.53%), benzoic acids and derivatives (2.53%), pyrimidines and pyrimidine derivatives (2.53%), pyridinecarboxylic acids and derivatives (2.53%), flavones, fatty alcohols (2.53%), fatty acids and conjugates (3.80%), purines and purine derivatives (5.06%), dicarboxylic acids and derivatives (5.06%), amines (5.06%), carbohydrates and carbohydrate conjugates (8.86%), and amino acids, peptides, and analogues (26.58%; Figure 1, Supplementary Table S1).

### Plant Growth Stimulation With *B. velezensis* S3-1

The Pak choi seeds did not germinate under the condition of 100% concentration. Compared with no inoculation, the shoot length of Pak choi did not change significantly at the 10% concentration, but the root length was severely inhibited and grew only one-fifth of the control. There was no significant difference in root length and shoot length of inoculated bacteria at the 4 and 2% concentrations compared with the non-inoculated bacteria. The difference in shoot length between the 1% concentration and the non-inoculated bacteria was not significant, but it could effectively promote the root length of the plant and increase the root length by about 56% (Supplementary Figure S1). This indicates that *B. velezensis* S3-1 had the ability to promote plant root growth and the potential to promote plant growth (Supplementary Figure S1). In view of these results, the 1% bacterial concentration was used for subsequent experiments.

### Plant Physiology and Soil Sample Analysis

ANOVA tests showed that the CFB treatment significantly decreased the hydrogen peroxide content (*p* < 0.05) compared with the S treatment, and significantly increased the soluble solid content and ascorbic acid of Pak choi (*p* < 0.05) compared with the CK treatment (Table 2). CFB treatment could significantly increase the soluble protein and ascorbic acid content relative to the CK and S treatments (*p* < 0.05), but the results showed that there was no significant difference in soluble sugar, nitrate content, and plant biomass between the CK, S, and CFB treatments (Table 2).

**Table 2:** Differences in hydrogen peroxide, Pak choi quality, and plant biomass between S, CFB, and CK treatment.

Sample name	Hydrogen peroxide content (μmol/g)	Soluble protein content (μg/ml)	Soluble solid content (°Bx)	Titratable acidity content (%)	Soluble sugar content (μg/ml)	Ascorbic acid (mg/100 g)	Nitrite content (μg/ml)	Biomass (g/plant)
CK	0.0379 ± 0.0118^a^	0.02848 ± 0.0000166^b^	2.33333 ± 0.127^b^	0.3 ± 0.130^b^	0.00881 ± 0.0000632^a^	18.7 ± 1.742^b^	6.407 ± 5.527^a^	6.88 ± 5.142^a^
S	0.0185 ± 0.00124^b^	0.0281 ± 0.0000691^c^	2.9 ± 0.1^a^	0.1125 ± 0.023^c^	0.00926 ± 0.000270^a^	22.033 ± 2.516^a,b^	5.0931 ± 4.224^a^	7.00633 ± 2.822^a^
CFB	0.0180 ± 0.00248^b^	0.02873 ± 0.0000230^a^	2.93333 ± 0.115^a^	0.45 ± 0.0001^a^	0.00885 ± 0.000157^a^	26.7 ± 1.732^a^	3.779 ± 1.202^a^	6.45367 ± 1.076^a^

Data are expressed as the average ± SD. Different letters in the same column denote statistically significant differences at *p* < 0.05.

Inoculation with CFB significantly decreased the pH and Fe content of the soil, and significantly increased EC and the P content (*p* < 0.05). However, inoculation with S treatment significantly decreased TN and sob (*p* < 0.05). Furthermore, inoculation with CFB or S treatment could significantly improve the length, tips, and forks of the plant roots (*p* < 0.05; Table 3).

**Table 3:** Differences in below-ground in S, CFB, and CK treatments.

Treatment	Soil							
	pH	EC (μs cm^−2^)	TN (g kg^−1^)	TC (g kg^−1^)	Fe content (mg kg^−1^)	Mg content (mg kg^−1^)	Na content (mg kg^−1^)	P content (mg kg^−1^)
S	7.357 ± 0.163^a^	985.333 ± 47.374^b^	0.103 ± 0.0035^a^	1.619 ± 0.057^b^	155.105 ± 4.355^a^	72.525 ± 4.243^a^	66.740 ± 46.600259^a^	133.913 ± 1.186^c^
CFB	6.680 ± 0.213^b^	1976.333 ± 47.962^a^	0.108 ± 0.0068^a^	1.940 ± 0.200^a^	139.7486 ± 9.741^b^	79.387 ± 17.776^a^	135.149 ± 60.689^a^	310.673 ± 21.102^a^
CK	7.540 ± 0.125^a^	932.667 ± 51.033^b^	0.112 ± 0.0107^a^	2.176 ± 0.184^a^	148.699 ± 3.4652^a^	68.919 ± 0.804^a^	138.660 ± 40.293^a^	195.517 ± 1.851^b^
**Treatment**	**Microbial diversity index**			**Plant root**				
	**SOB**	**Shannon**	**Simpson**	**Length(cm)**	**Root volume(cm^3^)**	**Tips**	**Forks**	
S	1919.667 ± 63.799^b^	6.327 ± 0.0750^a^	0.00550 ± 0.000572^a^	113.086 ± 17.319^a^	2.819 ± 1.567^a^	1555.333 ± 812.775^a^	4569.333 ± 1157.235^a^	
CFB	2029.000 ± 6.083^a^	6.463 ± 0.0925^a^	0.00475 ± 0.00167^a^	88.016 ± 16.193^a^	1.613 ± 1.012^a^	1121.333 ± 153.526^a^	3312.333 ± 481.531^a^	
CK	1964.667 ± 52.501^a^	6.362 ± 0.122^a^	0.00683 ± 0.00258^a^	39.0160 ± 5.219^b^	0.940 ± 0.191^a^	99.681 ± 57.551^b^	1632.000 ± 503.027^b^	

Data are expressed as the average ± SD. Different letters in the same column denote statistically significant differences at *p* < 0.05.

### Variations in Bacterial Composition

**Figure 2:**
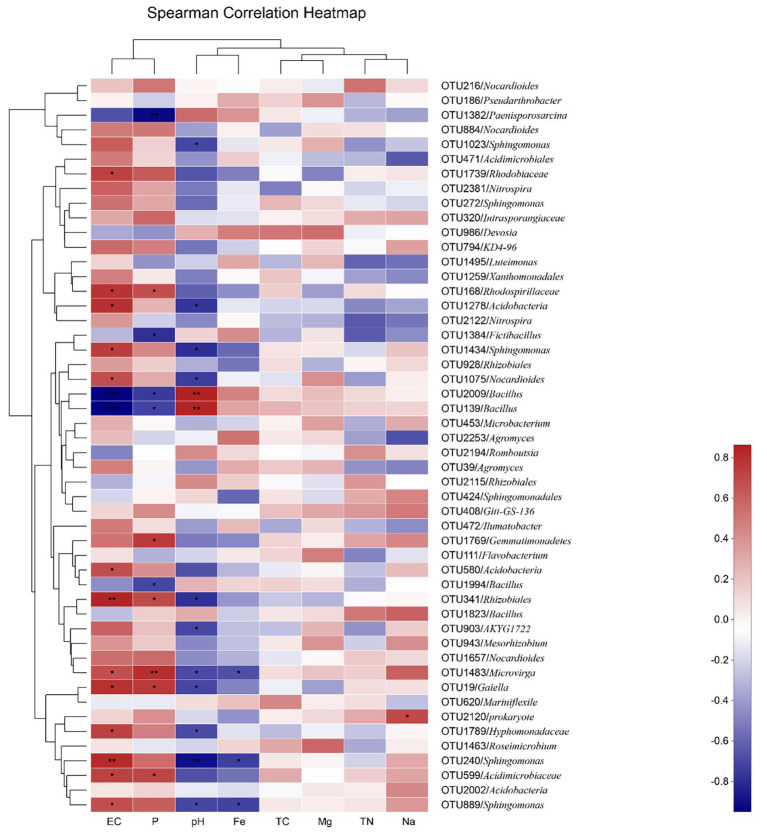
The heatmap depicts the relative abundances of the top 50 operational taxonomic units (OTUs) and Spearman’s correlations between OTU abundances and soil properties. The top 50 OTUs based on the values of mean decrease in the Gini index are selected and only the ones with average relative abundances of >0.1% are shown. EC, Electrical Conductivity; P, phosphorus; Fe, iron; TC, total carbon; Mg, magnesium; TN, total nitrogen; and Na, Sodium.

At the OTU level, the effects of environmental factors can be divided into four groups; EC and P, pH and Fe, TC and Mg, and TN and Na. The effects of the EC and P concentrations were the most significant, and the effects of pH and Fe came second. TC, TN, Mg, and Na had no significant effect (Figure 2). Among the OTUs, the relative abundances of 13 OTUs affiliated of *Rhodobiaceae* (OTU1739), *Rhodospirillaceae* (OTU168), *Acidobacteria* (OTU1278 and OTU580), *Sphingomonas* (OTU1434, OTU889, and OTU240), *Nocardioides* (OTU1075), *Rhizobiales* (OTU341), *Microvirga* (OTU1483), *Gaiella* (OTU19), *Hyphomonadaceae* (OTU1789), and *Acidimicrobiaceae* (OTU599) were positively correlated with EC in a significant way. On the contrary, the relative abundance of *Bacillus* (OTU2009 and OTU139) was significantly negatively correlated with EC. The relative abundance of *Rhodospirillaceae* (OTU168), *Gemmatimonadetes* (OTU1769), *Rhizobiales* (OTU341), *Microvirga* (OTU1483), *Gaiella* (OTU19), and *Acidimicrobiaceae* (OTU599) was significantly positively correlated with P content. In contrast, the relative abundance of *FictiBacillus* (OTU1384) and *Bacillus* (OTU139, OTU2009, and OTU1994) was negatively correlated with P content. In addition, the relative abundance of *Bacillus* (OTU139 and OTU2009) was significantly positively correlated with pH, while the relative abundance of *Acidobacteria* (OTU1278), *Sphingomonas* (OTU1434, OTU240, and OTU889), *Rhizobiales* (OTU341), *Microvirga* (OTU1483), and *Hyphomonadaceae* (OTU1789) was negatively correlated with pH. In addition, the relative abundance of *Microvirga* (OTU1483) and *Sphingomonas* (OTU889 and OTU240) was significantly negatively correlated with the Fe content. In addition, the relative abundance of *Prokaryote* (OTU2120) was significantly positively correlated with the Na^+^ content (Figure 2).

### The Overall Change of Bacterial Community

**Figure 3:**
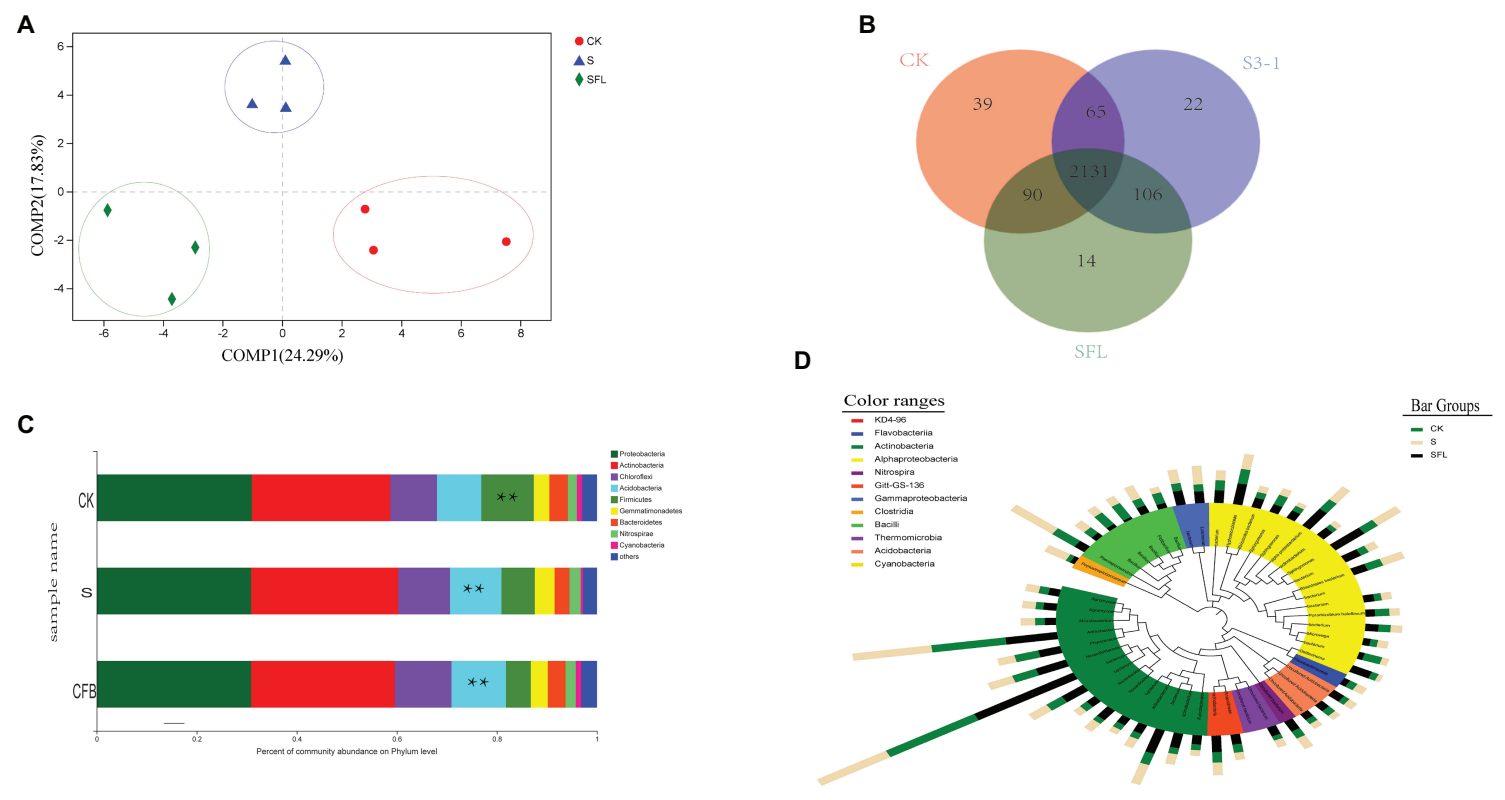
**(A)** Partial least square discriminant score plot of soil microbiota between the *Bacillus velezensis* strain S3-1 (S), cell-free fermentation broth (CFB), and no inoculation (CK) treatment. **(B)** The Venn diagram depicts OTUs that are shared or unique for different soil samples. **(C)** The relative abundance of bacterial taxa at the order level. Only the taxa with an average relative abundance of >0.1% are shown. **Represents a significant difference. **(D)** Top OTU members of the bacterial microbiome associated with the different treatments. Taxonomic dendrogram showing the core bacterial microbiome of each treatment. Color ranges identify phyla within the tree. Colored bars represent the relative abundance of each OTU in the different treatments. The taxonomic dendrogram was generated with one representative sequence of each OTU using R and displayed with the use of interactive tree of life (iTOL).

The purity, concentration, and integrity of DNA extraction could meet the requirements of high-throughput sequencing (Supplementary Table S2). The complete coverage of bacterial communities was confirmed by rarefaction curves (Supplementary Figure S2). Based on the OTU level, PLS-DA analysis found that S, CFB, and CK could be classified into one group. COMP1 explained 24.29% mutation rate, and COMP2 interpreted 17.83% mutation rate (Figure 3A).The phylogenetic breadth of the number of unique OTUs in the S and CFB treatments was lower than the CK treatment (Figure 3B). Fewer specific OTUs were found in the samples of the CFB treatments than the S treatments. It also showed that most OTUS were shared between samples (Figure 3B).

The main bacteria associated with the S and CFB treatments were the *Proteobacteria* and *Actinobacteria* phyla in the rhizosphere soil. The predominant of 16S rRNA sequences were clustered into *Proteobacteria* (30.9–31.56%), *Actinobacteria* (27.2–27.7%), and *Chloroflexi* (10.5–10.85%). The CFB treatment could decrease the relative abundance of *Firmicutes* and increase the relative abundance of *Acidobacteria* (*p* < 0.05). The S treatments could decrease the relative abundance of *Firmicutes* (*p* < 0.05; Figure 3C).

These results did not allow us to clearly understand which OTU changes lead to change at the phylum level, so we selected the top 50 OTUs to observe their distribution in the different treatment groups. They were divided into 11 genera, namely *Alphaproteobacteria* (24.46%), *Actinobacteria* (10.67%), *Clostridia* (1.51%), *Gammaproteobacteria* (3.94%), *Cyanobacteria* (1.31%), *Flavobacterium* (1.07%), *Thrmpmicrobia* (2.21%), *KD4-96* (3.49%), *Bacilli* (44.43%), *Nitrospira* (1.68%), and *Acidobacteria* (3.22%). The ANOVA model was (OTU) compartment which included all three treatments, followed by LSD. In the S treatment sample, OTU1384, OTU1495, and OTU2122 were significantly enriched, and OTU19 and OTU599 were significantly decreased (*p* < 0.05). In the CFB treatment, we observed a significant enrichment of OTU899, OTU240, OTU1769, OTU1789, OTU19, and OTU599, and a significant decrease of OTU1384 compared to the other treatments (*p* < 0.05). Compared with CK, S, and CFB treatments significantly decreased OTU139 and increased OTU1278 and OUT1075 (*p* < 0.05; Figure 3D).

At the same time, we found that the relative abundance of OTU1061 in the rhizosphere soil of the S treatment was six times higher than that of the CK and CFB treatment, and the 16S rDNA sequence of OTU1061 and *B. velezensis* S3-1 had 100% similarity (Supplementary Figure S3).

**Figure 4:**
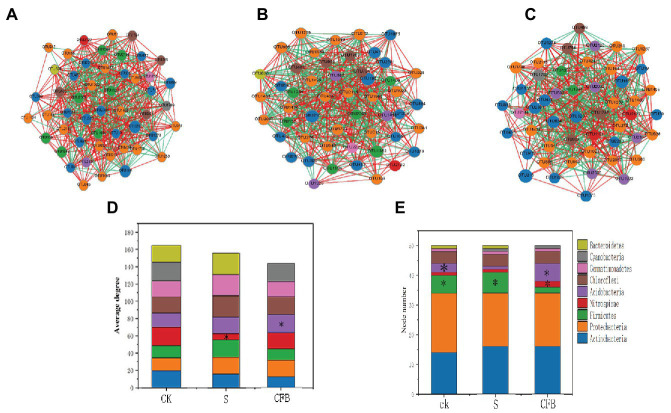
Network analyses of bacterial communities at CK treatment **(A)**, S treatment **(B)**, and CFB treatment **(C)**. Average degree between different treatment groups **(D)**. Node number between different treatment groups **(E)**. The nodes in the networks are colored by module class. *Represents a significant difference.

All three networks of different treatments clearly showed different compositions. For example, the inoculation with S treatment of *Bacteroidetes* (OTU620) had more negative correlations with other OTUs compared to the CK treatment. Furthermore, *Bacteroidetes* (OTU620) disappeared when inoculated with CFB treatment (Figures 4A–C). In addition, the relative abundance of *Actinomycetes* treated with CFB inoculation was increased, but the correlation was weaker compared to those in the CK and S treatments (Figures 4A–D). Moreover, compared with CK and S treatment, the number of *Acidobacteria* nodules inoculated with CFB treatment was higher (Figure 4E).

### Soil Enzyme and 16S Functional Prediction Analysis

**Figure 5:**
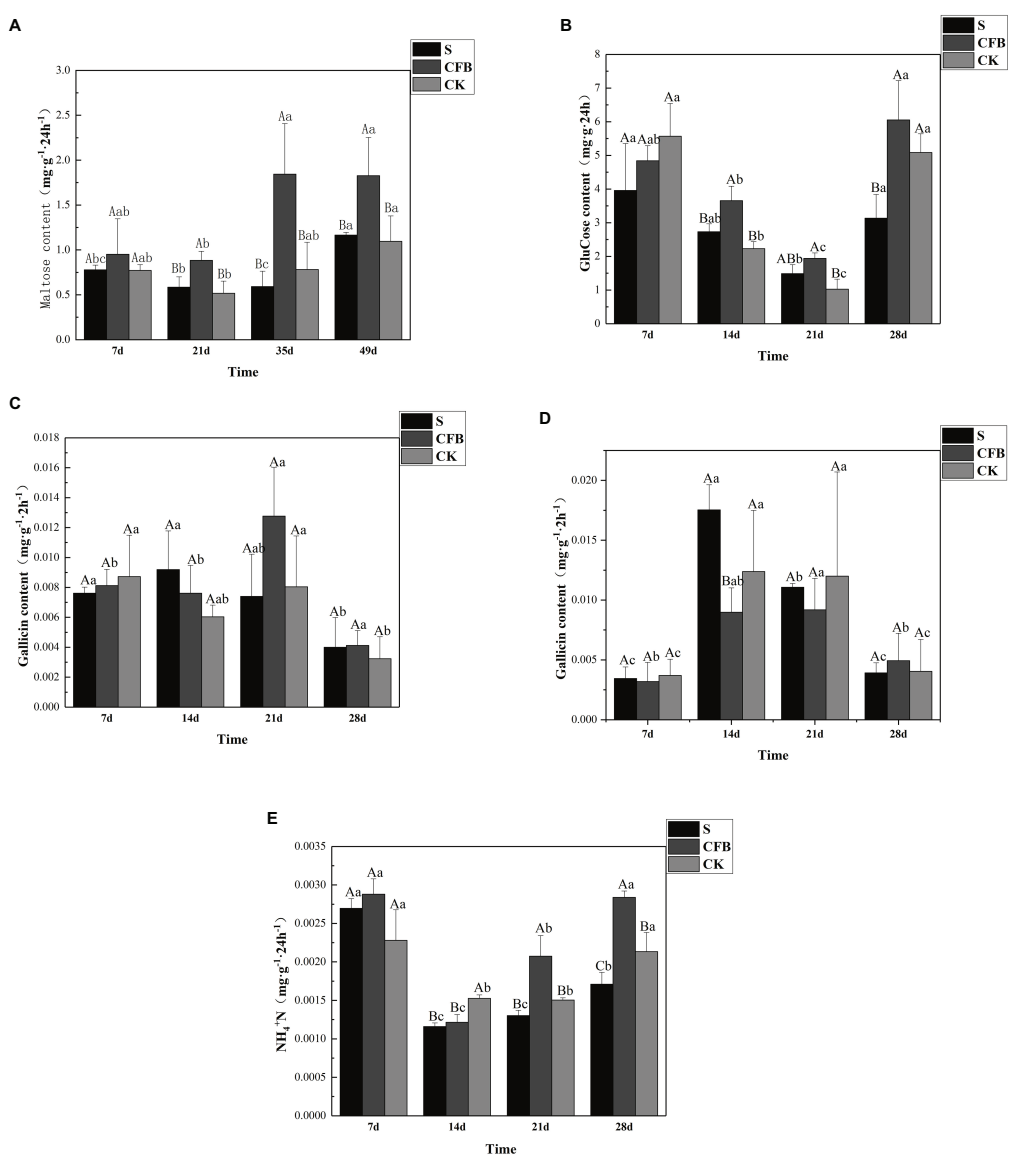
Activities of **(A)** amylase, **(B)** surcease, **(C)** polyphenol oxidase, **(D)** peroxidase, and **(E)** urease in the inoculated S, inoculated CFB, and CK treatment. The capital letter means different treatment at the same time; the lowercase letter means different treatment at different times. Data shown are mean ± SE, *n* = 3 replicates. Different letters indicate significant differences for each enzyme among treatments (one-way ANOVA, *p* < 0.05).

Our results showed a consistent trend in enzyme activity over time after inoculation with the S, CFB, and CK treatments. Compared with CK treatment, CFB treatment significantly increased the activities of amylase and sucrase on the 21st day, amylase, sucrase, and urease activities on the 35th day, and urease and sucrase activities on the 49th day (Figures 5A,B,D). However, the CFB treatment had no significant effect on the activity of polyphenol oxidase and peroxidase at any time (Figures 5C,D).

**Figure 6:**
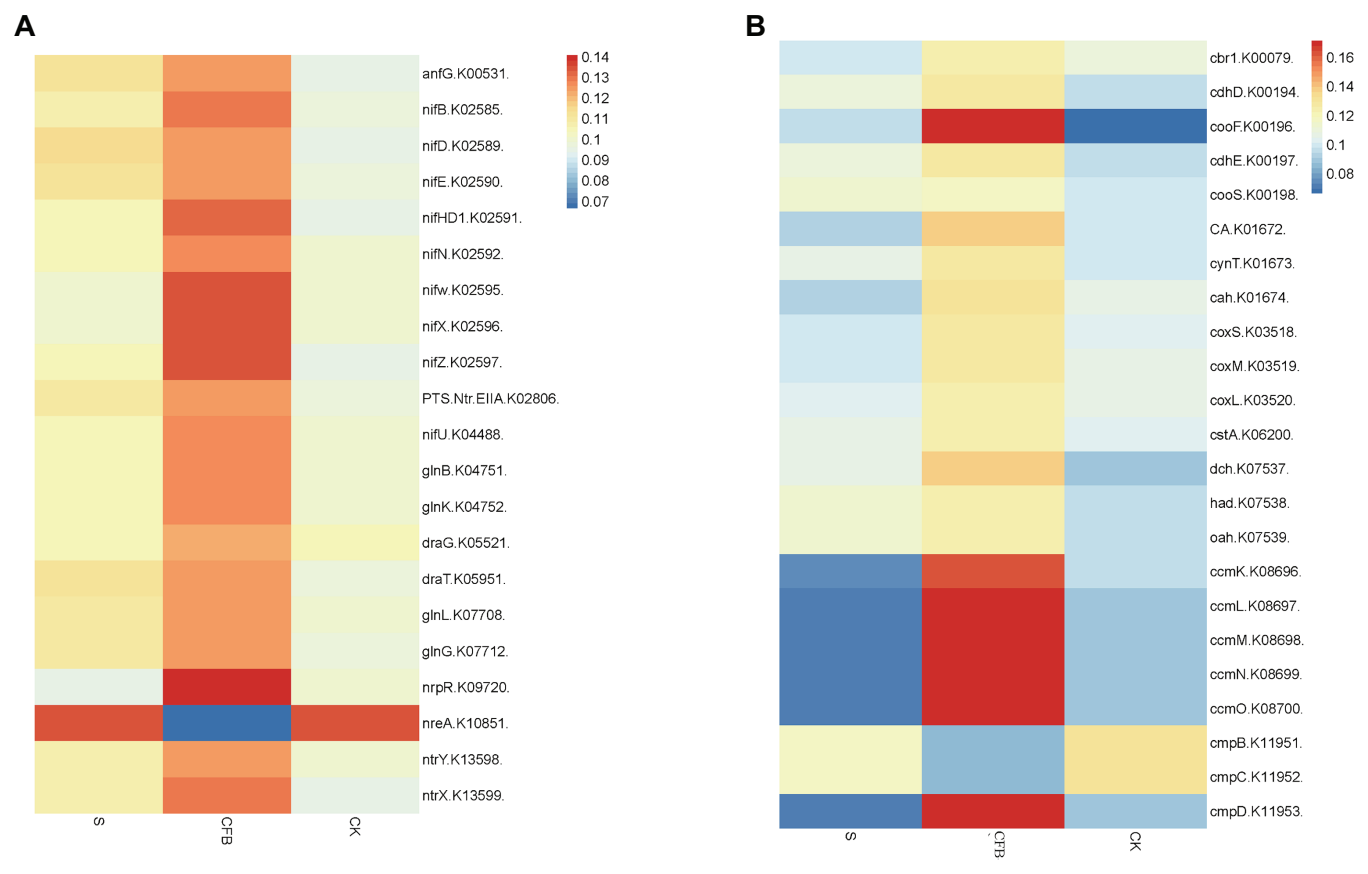
Potential gene abundance and potential function as it is based on 16S sequences. **(A)** The relative gene abundance associated with N metabolism. **(B)** The relative gene abundance associated with C metabolism.

The gene abundance was calculated by 16S sequences function prediction, the predicted values were all less than 0.17, indicating that the results were credible (Supplementary Table S3; Langille et al., 2013). Here was an increase in the relative abundance of genes associated with the nitrogen metabolism in addition to *nreA* (nitrate regulatory element) after CFB treatment compared with the S and CK treatments (Figure 6A). In addition to *cmpB* (Calvin cycle) and *cmpC*, the relative abundance of genes associated with carbon metabolism was also increased compared with S and CK treatments. Notably, the relative abundance of the *ccmL*, *ccmM*, *ccmN*, and *ccmO* genes was increased by more than 100% (Figure 6B).

## Discussion

In this study, we examined the effects of the S, CFB, and CK treatments on rhizosphere nutrient quality and soil properties (i.e., soil enzyme activity and soil microbial structure) of Pak choi.

### Identification of Fermentation Products

After the fermentation of *B. velezensis* S3-1, the composition of LB medium changed significantly. A large number of acids, alcohols, and sugars appeared after fermentation. Among them, the acids could reduce the pH of the rhizosphere soil. The addition of sugars will increase the C metabolism capacity in the soil, change the root structure of the plant, as well as increase the soluble solids, ascorbic acid, and antioxidant capacity (Figure 1, Supplementary Table S1; Shen and Bartha, 1997; Ya-Jie et al., 2018). The fermentation broth of *B. velezensis* S3-1 contains a variety of plant growth promoting substances, such as putrescine, spermidine, 2,3-butanediol, trigonelline, melatonin, etc. (Supplementary Table S1). These substances can activate plant genes and regulate the distribution of ions in plants, thus promoting plant growth (Seiler and Raul, 2005; Igarashi and Kashiwagi, 2010).

### Characterization of Bacteria and Plant Growth Under Laboratory Conditions

Inoculation with high concentration of bacteria (≥ 2%) did not promote the growth of plant roots and buds, while the inoculation with lower concentration (1%) promoted the growth of roots and shoots. This is due to the dual effects of IAA on inhibiting the growth of high concentration plants and promoting the growth of low concentration plants. ACC deaminase is an extracellular enzyme secreted by bacteria to promote plant growth. Siderophore and phosphorus-solubilizing activities, which are ensured by bacteria, also promote plant growth and improve plant quality (Podile and Kishore, 2006; Shaharoona et al., 2008; Hassen et al., 2018). However, the production of IAA requires the use of plant-derived tryptophan as a precursor for bacterial synthesis (Duca et al., 2014). S treatment can provide the above four substances, but the CFB treatment can only provide three other than IAA. This may explain why the S treatment is more likely to increase plant root length than the CFB treatment, although it is not significant (Table 2).

### Effects of Different Treatments on the Quality and Physiology of Pak Choi

Soluble sugar, soluble protein, titratable acid, and soluble protein are important flavor substances in vegetables (Cao et al., 2007). Compared with the other treatments, the CFB treatment enhanced the soluble protein of Pak choi (Table 2). This may be due to the increase in the EC of the soil (Durian et al., 2016). Interestingly, the CFB treatment did not increase the hydrogen peroxide content of the plants, but we observed a significant increase in the ascorbic acid (ASA) content, which may be resulted from the use by the plants of the ASA-GSH cycle pathway to scavenge hydrogen peroxide. Compared with other treatments, the CFB treatment resulted in the highest increases in titratable acid due to the Pak choi antioxidant properties (Table 2).

Compared with CK treatment, the number of root tips and forks of Pak choi were improved by CFB and S treatment, which were consistent with the research on the effect of PGPR inoculation on root structure, which helped plants enhance root tips, roots and absorb water and nutrients from the soil (Table 3; Moubayidin et al., 2009). In short, this means that the CFB treatment can effectively improve the nutritional quality of Pak choi, because it is resistant and could enhanced the absorption capacity of plants.

### Relationship Between Soil Properties and Bacterial Communities

The CFB treatment significantly changed the soil properties, because compared with other treatments; it reduced soil pH and Fe content, and increased P content and EC content. According to published research, increasing soil nitrogen cycling will reduce soil pH. The abundance of genes related to N cycle increased significantly in the CFB treatment group, which was a factor in the decrease of soil pH (Guo et al., 2010). The plant roots can absorb P to release OH^−^ to neutralize H^+^ in the soil to increase the soil pH. However, the accumulation of the P in the CFB-treated soil may be due to the absorption of P by plants. As a consequence, OH^−^ was not released, which was also responsible for the decrease in soil pH (Gardner and Boundy, 1983). It is believed that the reduction of soil pH will make the iron ions in the soil more easily absorbed by plants, which may be the reason for the decrease of iron ion content in the soil observed in the CFB treatment (Table 3; Guo et al., 2010). In short, due to the change of soil properties, compared with S and CK treatments, CFB treatment can significantly improve the nutritional quality of Pak choi.

Among the top 50 of the relative abundance, we observed that the *Acidobacteria* and *Bacillus* were most closely related to environmental factors. EC and pH can significantly affect the distribution of *Acidobacteria* in the soil (Figure 2). This is consistent with the results, we observed at the Phylum level (Figure 3C). Notably, the relative abundance of *Acidobacteria* increased with the decrease of pH, which was consistent with the strong correlation between the abundance of these bacteria and soil pH (Jones et al., 2009). The distribution of *Bacillus* was affected by the contents of EC, pH, and P, which was consistent with the study of Fierer (2017). This indicated that soil properties can drive changes in rhizosphere soil microbial communities, which may be an essential way to improve the nutritional quality of Pak choi.

### Soil Microbial Community Structure

*B. velezensis* S3-1 was capable of colonizing in rhizosphere soil (Supplementary Figure S3). Based on the PLS-DA, it ignored the random differences within the group and highlights the differences between the groups. We found that the S, CFB, and CK treatments could be clearly distinguished and divided into three groups (Figure 3A). This indicated that there were significant differences in soil microbial composition among the three treatments (Zhao et al., 2017). The results of the Venn diagram showed that compared with CK treatment, CFB treatment had fewer unique OTUs than S treatment (Figure 3B).

Our study found a significant increase in *Acidobacteria* under the CFB treatment (Figure 3C). *Acidobacteria* play a critical role in soil material circulation and ecological environment construction (Hugenholtz et al., 1998; Barns et al., 1999). In previous studies, the enrichment in Fe also increased the abundance of *Acidobacteria*, but our study found that there was no significant correlation between the distribution of *Acidobacteria* and Fe (Lauber et al., 2008; Da Rocha et al., 2010). Consistent with the results of S treatment, previous studies have shown that PGPR inoculation can increase the number of *Acidobacteria* in soil, thus promoting plant growth (Kalam et al., 2017). However, the CFB treatment could increase the abundance of *Acidobacteria* and promote plant growth compared to the S treatment (Figure 3D). The relative increase of *Acidobacteria* content also means that the ability to degrade residues in soil is enhanced, and the ability to metabolize carbon is enhanced (Pankratov et al., 2008; Pankratov and Ivanova, 2011). In short, the increase of *Acidobacteria* may be one of the key factors to promote the nutritional quality of Pak choi.

Since the main component of the *Firmicutes* is the *Bacillus* spp., the relative abundance of this group may be related to the reduction of *Bacillus* spp. The relative abundance of the *Firmicutes* in the CFB treatment was significantly lower than that in the S and CK treatments. In the phylogenetic trees of the top 50 OTUs of the three treatments, the relative abundance of *Bacillus* in the CFB treatment was lower than that in the CK and S treatments (Figure 3D). This meant that reducing natural soil *Bacillus* may help to improve the nutritional quality of Pak choi.

In the network, we observed that the node number of the *Acidobacteria* is increased in the CFB treatment. Compared with other treatments, the location of *Acidobacteria* tended to the core, which meant that *Acidobacteria* played an important role among bacterial communities (Figures 4A–C). At the same time, the number of nodes in *Firmicutes* decreased, which meant that the relative abundance of *Firmicutes* decreased, so it is more difficult to enter top 50 OTUs.

### Prediction and Analysis of Soil Enzyme and 16S Function

C cycle and N cycle are very important in crop growth. The *cmp* gene family, *ccm* gene family, and *cox* gene family are responsible for carbon capture, carbon sequestration, and oxidation of CO to CO_2_. Compared with other treatment groups, the CFB treatment group could increase the abundance of the *cmpD* (The low−CO_2_ high affinity HCO_3_ – transporter) gene, *ccm* gene family, and *cox* (carboxydotrophic Oligotropha carboxidovorans) gene families. This means that the ability of soil microorganisms for carbon capture, carbon sequestration, and CO to CO_2_ oxidation has been increased (Figure 4A; John et al., 2007; Heinrich et al., 2018; Willis et al., 2019).

*AnfG* (alternative nitrogenase) gene, *nif* (N_2_-fixing) gene family, *gln* (*PII signal transduction*) gene family, *dra* (nitrogenase reductase ADP-ribosyl *transferase*) gene family, and *ntr* gene family are generally responsible for regulating nitrogenase activity, N metabolism, dinitrogenase reductase activity, and nitrate metabolism. Compared with the S and CK treatments, the CFB treatment increased the abundance of the above gene families, which means that the ability of soil microorganisms to fix nitrogen, nitrogen metabolism, and nitrate metabolism was enhanced (Figure 4B; Greco et al., 2012; Bonato et al., 2016; Moure et al., 2019).

The enzyme activity is an important indicator to measure soil fertility, especially invertase, amylase, catalase, peroxidase, and urease (Henry et al., 2005; Allison and Treseder, 2008). This was also supported by our research. The activities of invertase, amylase, and urease showed an up-down-up pattern in the three different treatment groups. Compared with the other treatments, the activities of invertase, amylase, and urease in CFB treatment were significantly increased (*p* < 0.05), but the peroxidase and catalase activities were not increased (Figures 5A–E). This indicated that compared with the CK and S treatment, the CFB treatment enhanced the C and N cycling in soil, which was consistent with our prediction results using PICRUS. Therefore, the change of enzyme activity was caused by the change of soil microbial community (Marumoto et al., 1982; Singh et al., 1989).

In summary, CFB can improve the quality of Pak choi, which may be explained by three main factors. Firstly, the CFB treatment has substances that can directly promote plant growth and improve the plant quality, such as ACC deaminase, siderophores, phosphorus-solubilizing activity, and sugar. Secondly, CFB treatment changed soil properties and indirectly improved the nutritional quality of Pak choi by increasing soil nitrogen cycling and acid production. Finally, soil characteristics driven the changes of rhizosphere microbial community, that is, the content of acid bacteria increased greatly, and the number of protobacillus decreased, which further enhanced the nutritional quality of Pak choi.

However, our experiments still have some shortcomings. Due to the complexity of soil microbes, we were unable to effectively identify which microorganisms significantly enhance the N cycle and thus reduce the soil pH. At the same time, due to the complexity of bacterial metabolites, it is not possible to determine which substance can attract acidic bacteria or other bacterial communities in soil.

Considering that bio-fertilizers are often ineffective due to competition for resources and soil complexity with natural microbiota, it is encouraging to find that a CFB can also improve the quality of crops. This discovery provides a new idea for the future application of CFB in agriculture field.

## Data Availability Statement

The datasets generated for this study can be found in the NCBI with accession numbers CP016371.1 and PRJNA534410.

## Author Contributions

KHL and QJ designed the experiment, and KHL completed most of the experiments. KHL and QJ co-authored the article. SSL, WWL, CHZ, JRJ, QJ and LCL did a small number of experiments and provided experimental methods. MX guided the experiment and modified the manuscript. All authors contributed to the article and approved the submitted version.

### Conflict of Interest

The authors declare that the research was conducted in the absence of any commercial or financial relationships that could be construed as a potential conflict of interest.

## Footnotes

^1^http://drive5.com/uparse/

^2^http://rdp.cme.msu.edu/
