# Supplementary material for: Cell-Free Fermentation Broth of Bacillus velezensis Strain S3-1 Improves Pak Choi Nutritional Quality and Changes the Bacterial Community Structure of the Rhizosphere Soil
Source: Front Microbiol. 2020 Sep 10;11:2043. doi: 10.3389/fmicb.2020.02043 (PMC7533579; doi:10.3389/fmicb.2020.02043)
Supplement: Supplementary file 1 [file Data_Sheet_1.docx]

**Cell-free fermentation broth of *Bacillus velezensis* strain S3-1 improves the quality of Pakchoi and changes the bacterial community structure of the rhizosphere soil**

Kaiheng Lu1*,Qing Jin1, Yibo Lin1,Wenwei Lu1,Songshuo Li1, Chenhao Zhou1,Jieren Jin1, Qiuyan Jiang1,Ming Xiao1*

College of Life Sciences, Shanghai Normal University, Shanghai, 200234, China

*Corresponding authors: Professor Ming Xiao

Mr. Kaiheng Lu,

college of Life Sciences Shanghai Normal University Shanghai, 200234

P. R. China

Tel: ++86-17621543755

Fax: ++86-(0)-21-65642468

E-mail addresses: [xiaom88@shnu.edu.cn](mailto:xiaom88@shnu.edu.cn)

[lukaiheng@otulook.com](mailto:lukaiheng@otulook.com)





**Fig. S1** Effect of inoculation of *B.velezensis* stain S3-1 on plant root length and shoot length. Different letters indicate significant differences for root or shoot among treatments.


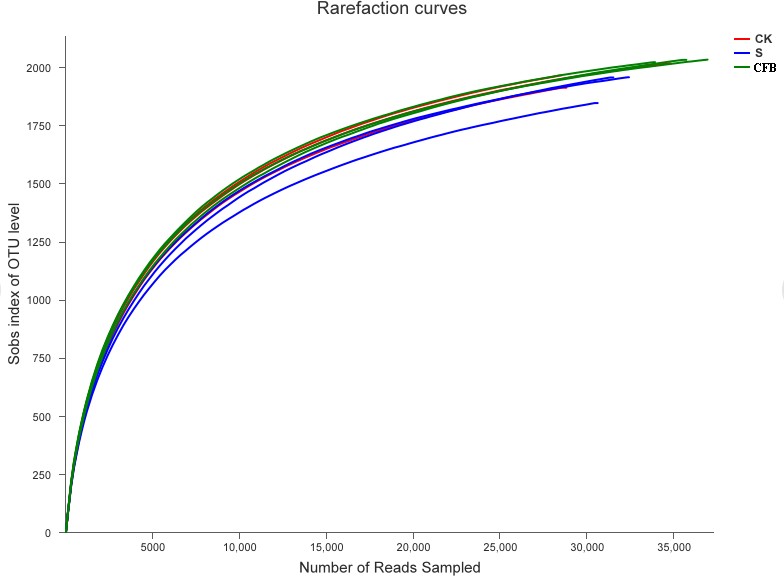


**Fig. S2** The rarefaction curve of each sample


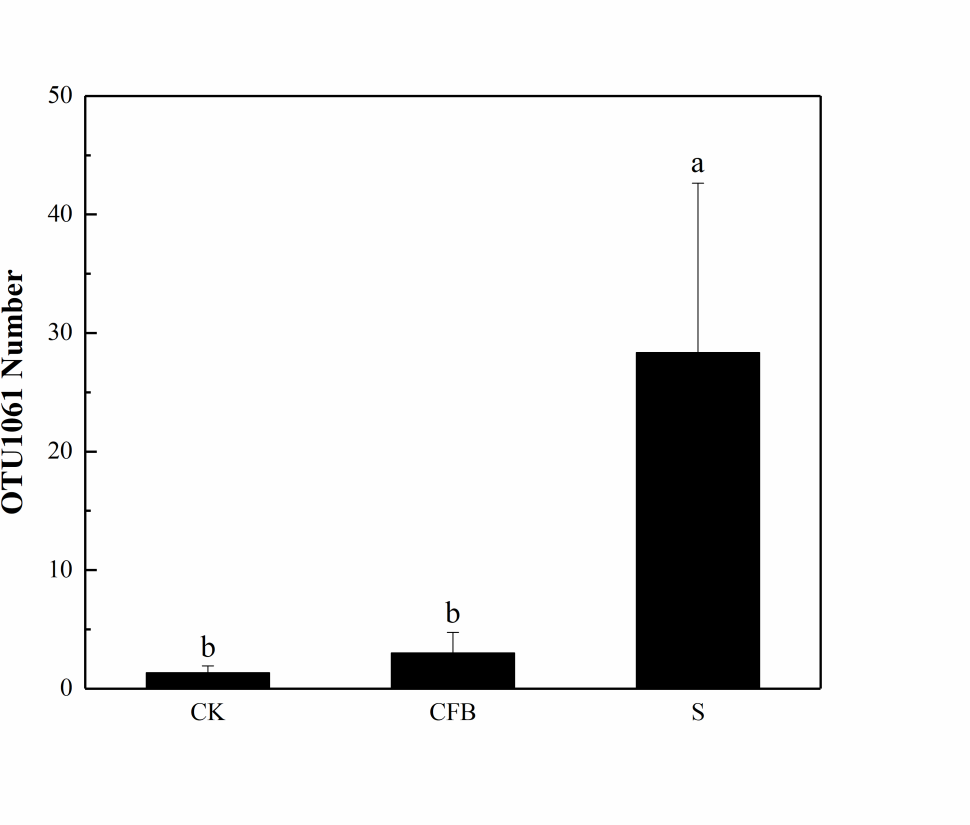


**Fig.S3** Observed number of OTU1061


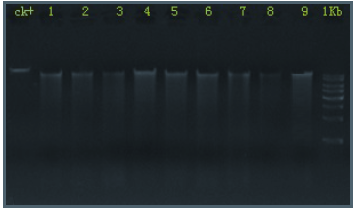


**Fig.S4** Results of DNA integrity test

**Table S1** Metabolites of *B. velezensis* S3-1

| Metabolite | Metabolite | Metabolite | Metabolite |
| --- | --- | --- | --- |
| L-glutamic acid | galactinol | maleic acid | trans-3-hydroxy-L-proline |
| S(-)willardiine | trigonelline | isoxanthopterin | 4-quinolinecarboxylic acid |
| glycine | mucic acid | 3-hydroxyanthranilic acid | L-lysine |
| L-methionine | dihydro-4,4-dimethyl-2,3-furandione | Methaqualone | valeramide |
| Cannabinol | nicotinic acid | 4-aminophenol | dibenzofuran |
| iminodiacetic acid | 2-hydroxybiphenyl | beta-glycerolphosphate | 3-methyl-L-histidine |
| L-allothreonine | 1-octanol | cycloleucine | L-proline |
| heptadecanoic acid | N-omega-acetylhistamine | 3-Hydroxy-3',4'-Dimethoxyflavone | prunetin |
| D-sphingosine | 4-O-Methylphloracetophenone | alpha ketoglutaric acid | threose |
| glycolic acid | 3,5-dihydroxyphenylglycine | 2,3-butanediol | D-mannitol |
| 1-methyl-L-tryptophan | L-threonine | tyrosine | cysteinylglycine |
| benzoquinone | 1,4-dihydroxy-2-naphthoic acid | psicose | butylbenzene |
| 22-hydroxycholesterol | threonic acid | pipecolic acid | DL-4-hydroxymandelic acid |
| Oxymorphone | adenosine'-monophosphate | melatonin4 | pantolactone |
| orcinol | 1-octadecene | epsilon-caprolactam | trans-resveratrol |
| 4-Acetoxyphenol | xanthosine | L-histidine | mandelic acid |
| beta-hydroxyisovalerate | 6-methyl-5-hepten-2-one | 2-furoic acid | 2-aminooctanoic acid |
| 2-Methoxyxanthone | benzoic acid | beta-ionone | cellotetraose |
| 7,4'-Dihydroxyflavone | glycerol | 2,3-dihydroxypyridine | 1,2,4,5-tetramethylbenzene |
| 3-aminopropionitrile | DL-3,4-dihydroxyphenyl glycol | serotonin | kestose |
| juglone | L-cysteine | spermidine | 5,6-dihydrouracil |
| citraconic acid | L-serine | N-carbamyl-L-glutamic acid | tyrosine methyl ester |
| malonic acid | 6-hydroxynicotinic acid | cyclohexanamine | 4-hydroxyphenylglycine |
| benzyl thiocyanate | 1,3-propanediol | DL-dihydrosphingosine | IS |
| orotic acid | carbazole | glycylproline | 2,6-diaminopimelic acid |
| Barbital | acetohydroxamic acid | 1,2-cyclohexanedione | 2-amino-2-methyl-1,3-propanediol |
| N-acetyl-5-hydroxytryptamine | L-pyroglutamic acid | N-acetyl-D-tryptophan | 5alpha-Cholestan-3beta-Ol-6-One |
| galactitol | 1-stearoyl-rac-glycerol | acetanilide | citric acid |
| porphine | fumaric acid | phytosphingosine | benzoylformic acid |
| 5-hydroxyindole-2-carboxylic acid | Oxazepam | tyramine | fructose |
| L-sorbose | D-Ala-D-Ala2 | trans-aconitic acid | 4-hydroxypyridine |
| DL-isoleucine | eicosapentaenoic acid | trans-4-hydroxy-L-proline | Apigenin |
| N-gamma-acetyl-N-2-formyl-5-methoxykynurenamine | 4-(2-hydroxyethyl)phenol | guanosine | benzene-1,2,4-triol |
| DL-4-hydroxy-3-methoxymandelic acid | 5-cholesten-3-beta-7-alpha-diol | 2-indanone | acetol |
| L-Phenylalanine | glyceric acid | glycerol-phosphate | 2-hydroxypyridine |
| methyl-beta-D-galactopyranoside | 8-aminocaprylic acid | L-mimosine | beta-myrcene |
| L-ornithine | trans,trans-muconic acid | 1-octen-3-ol | myo-inositol |
| 5-aminovaleric acid | D-allose | Fenfluramine | 5-Hydroxy-2',4',7,8-Tetramethoxyflavone |
| phytol | synephrine | oxalic acid | adenine |
| N-methylglutamic acid | 3,4',5,6,7-Pentamethoxyflavone | ethanolamine | 1-methylhydantoin |
| 5,6-dihydro-5-methyluracil | trans caftaric acid | Thebaine | 3-chloro-L-tyrosine |
| 4-vinylphenol | 2-piperidone | uric acid | ribitol |
| 5,6-dimethylbenzimidazole | neryl acetate | Antiarol | xylulose |
| benzyl isothiocyanate | maleamic acid | L-homocystine | saccharin |
| 2'-Deoxyadenosine 3':5'-cyclic monophosphate | 1-nonanol | 4-hydroxycinnamic acid | taxifolin |
| putrescine | hypoxanthine | 3-hydroxyflavone | cytosine |
| 5-methoxy-3-indoleacetic acid | O-benzyl-L-tyrosine | octopine | L-alanine |
| D-trehalose | 1-hexadecanol | 1,4-dideoxy-1,4-imino-D-arabinitol | trans-3-hydroxy-L-proline |
| L-norleucine | xanthine | sarcosine | 4-quinolinecarboxylic acid |

| Number | Sample name | Concentration(ng/μl) | OD260/280 | OD260/230 |
| --- | --- | --- | --- | --- |
| 1 | S1 | 36.60 | 1.90 | 0.08 |
| 2 | S2 | 50.60 | 1.85 | 0.14 |
| 3 | S3 | 40.60 | 1.81 | 0.07 |
| 4 | SF1 | 58.50 | 1.88 | 0.33 |
| 5 | SF2 | 53.30 | 1.89 | 0.11 |
| 6 | SF3 | 50.40 | 1.80 | 0.16 |
| 7 | CK1 | 44.60 | 1.85 | 0.17 |
| 8 | CK2 | 32.00 | 1.79 | 0.28 |
| 9 | CK3 | 36.30 | 1.94 | 0.06 |

**Table S2** DNA concentration between nine samples

| Sample name | CK1 | CK2 | CK3 | S1 | S2 | S3 | CFB1 | CFB2 | CFB3 |
| --- | --- | --- | --- | --- | --- | --- | --- | --- | --- |
| NIST | 0.122 | 0.135 | 0.155 | 0.153 | 0.131 | 0.134 | 0.129 | 0.119 | 0.138 |

**Table S3** NIST values of different treatment
